# Economic evaluation of an exercise-counselling intervention to enhance smoking cessation outcomes: The Fit2Quit trial

**DOI:** 10.1186/s12971-017-0126-y

**Published:** 2017-03-29

**Authors:** William Leung, Vaughan Roberts, Louisa G. Gordon, Christopher Bullen, Hayden McRobbie, Harry Prapavessis, Yannan Jiang, Ralph Maddison

**Affiliations:** 1 University of Otago, Wellington, PO Box 7343, New Zealand; 2 University of Auckland, Private Bag 92019, Auckland, New Zealand; 3 Berghofer Medical Research Institute, Brisbane, Australia; 4 UK Centre for Tobacco Control Studies, Wolfson Institute of Preventive Medicine, Barts & The London School of Medicine and Dentistry, Queen Mary University of London, London, UK; 5 School of Kinesiology, Faculty of Medical and Health Sciences, University of Western Ontario, 1151 Richmond St, London, ON Canada; 6 Centre for Physical Activity and Nutrition, Deakin University, Melbourne, VIC Australia

**Keywords:** Economic evaluation, Exercise, Smoking cessation, Randomised controlled trial

## Abstract

**Background:**

In the Fit2Quit randomised controlled trial, insufficiently-active adult cigarette smokers who contacted Quitline for support to quit smoking were randomised to usual Quitline support or to also receive ≤10 face-to-face and telephone exercise-support sessions delivered by trained exercise facilitators over the 24-week trial. This paper aims to determine the cost-effectiveness of an exercise-counselling intervention added to Quitline compared to Quitline alone in the Fit2Quit trial.

**Methods:**

Within-trial and lifetime cost-effectiveness were assessed. A published Markov model was adapted, with smokers facing increased risks of lung cancer and cardiovascular disease.

**Results:**

Over 24 weeks, the incremental programme cost per participant in the intervention was NZ$428 (US$289 or €226; purchasing power parity-adjusted [PPP]). The incremental cost-effectiveness ratio (ICER) for seven-day point prevalence measured at 24-week follow-up was NZ$31,733 (US$21,432 or €16,737 PPP-adjusted) per smoker abstaining. However, for the 52% who adhered to the intervention (≥7 contacts), the ICER for point prevalence was NZ$3,991 (US$2,695 or €2,105 PPP-adjusted). In this adherent subgroup, the Markov model estimated 0.057 and 0.068 discounted quality-adjusted life-year gains over the lifetime of 40-year-old males (ICER: NZ$4,431; US$2,993 or €2,337 PPP-adjusted) and females (ICER: NZ$2,909; US$1,965 or €1,534 PPP-adjusted).

**Conclusions:**

The exercise-counselling intervention will only be cost-effective if adherence is a minimum of ≥7 intervention calls, which in turn leads to a sufficient number of quitters for health gains.

**Trial registration:**

Australasian Clinical Trials Registry Number ACTRN12609000637246

## Background

Smoking cessation is an important public health challenge as it has intermediate and long-term health benefits. Treatments to aid smoking cessation that are widely available, accessible, and cost-effective would therefore have great potential for public health benefit.

Despite the availability of effective treatments for smoking cessation in many countries, the proportion of people who successfully quit smoking is disappointingly low [1, 2]. New approaches to sustain the implementation of effective smoking cessation treatments are needed. One option proposed as an aid for smoking cessation is the use of exercise.

A recent Cochrane review of exercise interventions for smoking cessation identified 20 randomised controlled trials (*n* = 5870) with adequate follow-up (ie, at least six months after the quit date) [3]. However, only 2 of the 20 trials offered evidence that exercise aided smoking cessation in the long term.

The authors of the Cochrane review highlighted a number of methodological issues that have limited previous research: small sample sizes, lack of statistical power, gender bias, lack of sufficient intensity or duration of the intervention to positively affect smoking outcomes, and reliance on structured supervised exercise regimens, which limits scalability. Moreover, there is a complete dearth of research to evaluate the cost-effectiveness of this approach. The Fit2Quit trial was conducted in an attempt to address some of these methodological concerns that were identified in an earlier version of the Cochrane review, and to determine the cost-effectiveness of an exercise-counselling intervention in addition to standard smoking cessation support to enhance abstinence rates compared to standard cessation support alone. The objective of this paper is to present the within-trial (at 24-week follow-up) and lifetime cost-effectiveness analysis (using a Markov model) of the Fit2Quit trial. Additionally, we sought to identify how cost-effectiveness would alter by investigating a subgroup of intervention participants who adhered well to the intervention, that is, those who may incur greater intervention costs but potentially have greater quit rates. This may be of use in directing future research and future counselling program improvement.

## Methods

### Trial and intervention summary

The full details of the design and conduct of the Fit2Quit trial have been published elsewhere [4]. In brief, it was a parallel group two-arm randomised controlled trial conducted in New Zealand (NZ) between 2009 and 2012. Participants were randomised at a 1:1 ratio to either receive an exercise intervention plus usual stop smoking support, or to usual stop smoking support alone (ie, Quitline). Table 1 shows the baseline characteristics of the trial participants (*n* = 906). There were no significant differences in socio-demographic, smoking and physical activity profiles at baseline between control and intervention groups. All enrolled participants were encouraged to set a quit date, and offered one-to-one Quitline telephone support for three months, as well as up to eight weeks of subsidised nicotine replacement therapy.

**Table 1:** Baseline characteristics for trial participants by group

	Control (*n* = 455)	Intervention adherent (*n* = 236)	Intervention non-adherent (*n* = 219)
Age (years), mean ± SD	37.3 ± 12.2	39.8 ± 12.4	35.2 ± 11.7
Sex, *n* (%)			
Male	207 (45.9)	105 (44.5)	103 (47)
Female	244 (54.1)	131 (55.5)	116 (53)
Prioritised ethnicity, *n* (%)			
NZ European	214 (47.5)	132 (55.9)	86 (39.3)
Māori	138 (30.6)	63 (26.7)	79 (36.1)
Pacific	55 (12.2)	15 (6.4)	32 (14.6)
Asian	11 (2.4)	5 (2.1)	8 (3.7)
Other	33 (7.3)	21 (8.9)	14 (6.4)
Number of cigarettes/day, mean ± SD	19.8 ± 9.2	20 ± 10.5	18.8 ± 8.1
Age of smoking onset (years), mean ± SD	15.5 ± 4.2	15.9 ± 4.9	15.1 ± 3.3
Years smoking continuously, mean ± SD	20.3 ± 11.9	21.7 ± 12.4	18.5 ± 11.5
Fagerström Test of Nicotine Dependence score, mean ± SD	5.6 ± 2	5.5 ± 1.9	5.5 ± 1.9
Previous quit attempts, *n* (%)			
Yes	348 (77.2)	194 (82.2)	174 (79.5)
No	103 (22.8)	42 (17.8)	45 (20.6)
Number of quit attempts in previous 12 months, *n* (%)			
Never attempted to quit before	103 (22.8)	42 (17.8)	45 (20.6)
None	204 (45.2)	113 (47.9)	89 (40.6)
One	83 (18.4)	43 (18.2)	50 (22.8)
Two	28 (6.2)	19 (8.1)	18 (8.2)
Three	14 (3.1)	11 (4.7)	4 (1.8)
Four or more	18 (4)	8 (3.4)	12 (5.5)
Do not know	1 (0.2)	0 (0)	1 (0.5)
Confidence to quit, *n* (%)			
One (very low)	8 (1.8)	2 (0.9)	5 (2.3)
Two	15 (3.3)	5 (2.1)	7 (3.2)
Three	97 (21.5)	30 (12.7)	53 (24.2)
Four	140 (31)	76 (32.2)	72 (32.9)
Five (very high)	190 (42.1)	123 (52.1)	81 (37)
Missing	1 (0.2)	0 (0)	1 (0.5)
Physical activity (MET minutes/week), mean ± SD			
Leisure time domain	601 ± 1,150	498 ± 799	621 ± 1,343
Work domain	3,557 ± 6,370	3,982 ± 6,532	4,015 ± 7,096
Active transport domain	437 ± 1,047	338 ± 1,025	531 ± 1,720
Domestic and garden domain	1,838 ± 2,877	1,665 ± 2,218	1,930 ± 3,032
Total walking	1,769 ± 2,838	1,844 ± 2,706	1,634 ± 2,534
Total moderate physical activity	3,191 ± 3,728	3,030 ± 3,460	3,624 ± 4,453
Total vigorous physical activity	1,472 ± 3,728	1,643 ± 4,008	1,832 ± 4,316
Total physical activity	6,481 ± 7,226	6,489 ± 7,389	7,132 ± 8,325

*MET* Metabolic Equivalent of Task

Intervention group participants (*n* = 455) commenced a six-month home and community-based exercise programme delivered by Green Prescription (GRx) services, delivered in 10 contacts (face-to-face and telephone support sessions) over six months. GRx involves a referral from primary care to agencies that support physical activity [5]. In this trial however, participants randomised to the intervention group were referred by the researcher, without involving a General Practitioner. Once referred, trained exercise-facilitators (participant-support person [PSPs]) contacted participants and offered telephone counselling to promote and support exercise behavior. PSPs encouraged participants to work towards a goal of partaking in ≥30 min of moderate-vigorous aerobic-based exercise on most days of the week, in line with the 1996 US Surgeon General’s recommendations [6].

The Fit2Quit trial results have been reported elsewhere [7]. Smoking abstinence rates at 24 weeks were moderately high, but not statistically significantly different between intervention and control groups. Of the 455 participants who were randomised to the intervention group, 52% (*n* = 236) completed at least seven of the ten intervention calls (median number of calls = 7, interquartile range = 4─9), hereafter the adherent intervention group. When compared with the control group, a significant treatment effect on smoking cessation was found for those who were in the adherent intervention group (OR 0.67, 95% CI 0.46 to 0.98, *p* = 0.04). Therefore, the economic evaluation using a *lifetime horizon* is focused on this adherent intervention group.

### Outcomes data

Follow-up assessments were completed at 8 and 24 weeks after the nominated quit date. The primary outcome was self-reported point-prevalence (ie, not a single puff of a cigarette in the past seven days) at 24 weeks after the nominated quit date. At baseline and 24 weeks, self-reported physical activity levels were measured with the International Physical Activity Questionnaire – Long Form [8].

Health-related quality of life (HRQoL) was assessed using the EQ-5D questionnaire with utility values obtained from NZ tariff 2. Perfect health and death are anchored at utilities of 1 and 0, respectively. ‘Imperfect’ health is valued at less than 1. The time spent at a specific utility was used to generate a quality-adjusted life-year (QALY). For example, if one year with advanced stage lung cancer has a utility of 0.56, then half a year with that disease is equivalent to 0.28 QALYs.

Table 2 details the key parameters used in the model. Gender-specific continuous abstinence rates and health-state utility values (for the ‘well and smoking’ states) have been taken from the trial data. Epidemiological data were sourced from NZ life tables, disease incidence rates, national databases and international literature.

**Table 2:** Data parameters in model

Model parameter	Mean (se)		Distribution	Source
	Male	Female		
Smoking				
24-week point prevalence rates				
a) intervention – adherent group (*n* = 236)	35.2% (4.7%)	29.8% (4.0%)	Beta	Trial
b) usual care	24.2% (3.0%)	19.7% (2.5%)	Beta	Trial
Relapse rate from 24 weeks (end of trial) to 12 months	21%	^a^		[25]
Relapse rates after 12 months	30% cumulative	^a^		[26]
Lung cancer				
Annual incidence	eg 0.00180^b^	eg 0.00165^b^	Beta	[27]
Proportion – early stage lung cancer (I&II)	20%	^a^		[14]
Proportion – adv stage lung cancer (III&IV)	80%	^a^		[14]
Relative risk of lung cancer in ex-smokers vs. general population	eg 1.771	^a^		[14]
	15 years after quitting^b^			
CVD				
Annual incidence	eg 0.03095^b^	eg 0.01843^b^	Beta	[28]
Relative risk of CVD in smokers vs. general population	1.42 (0.031)	^a^	LogNormal	[29]
Relative risk of CVD in ex-smokers vs. smokers	0.71 (0.036)	^a^	LogNormal	[29]
Mortality				
a) pre-hospital death given a CVD event	18.1%	^a^		[30]
b) post-hospital death given a CVD event (≤28 days)	7.1%	^a^		[30]
c) background mortality (annual)	eg 0.01071^b^	eg 0.00715^b^	Beta	[31]
Utility scores				
a) well (no lung cancer or CVD)				
baseline	0.800	^a^		Trial
continue smoking after end of trial	0.800	^a^		Assumption
abstain from smoking at end of trial	0.830	^a^		[11]
b) early stage lung cancer	0.73 (0.020)	^a^	Beta	[14]
adv stage lung cancer	0.56 (0.043)	^a^	Beta	[9]
c) weighted average CVD	0.611			[9, 10]
Excess health system costs (NZ$)				
a) lung cancer, first year of diagnosis	eg 23,970^b^	eg 22,256^b^	Gamma	[13]
lung cancer, subsequent annual costs before death	eg 5,375^b^	eg 4,341^b^	Gamma	[13]
lung cancer, last six months before cancer death	eg 16,615^b^	eg 20,300^b^	Gamma	[13]
b) CVD, first year of diagnosis if hospitalised	eg 11,327^b^	eg 10,189^b^	Gamma	[13]
CVD, last six months before CVD death	eg 17,573^b^	eg 11,048^b^	Gamma	[13]

NZ$1 = US$0.68 = €0.53

^a^Same value for both sexes

^b^Age/time-dependent values used in tables. If no other details are given, then the example is for a 65-year-old individual

Health-state utilities for cardiovascular disease (CVD) were taken from Sullivan et al. [9] where community-based UK preferences were applied to EQ-5D descriptive questionnaire responses in the US-based Medical Expenditure Panel Survey (*n* = 79,522); coronary artery disease (0.629), cerebrovascular disease and stroke (0.649), congestive heart failure (0.493), and peripheral vascular disease (0.657). Each disease state was then weighted by their proportional incidence in the National Minimum Dataset (NMDS) [10] of NZ CVD hospital admissions, respectively 54.9, 21.9, 17.6, and 5.6%, to give a composite CVD utility of 0.611.

It was assumed that the utility after quitting smoking, and being CVD and lung cancer free, would improve by 0.03: Tillman and Silcock [11] noted this difference between smokers (0.75) and ex-smokers after 5 years abstinence (0.78) using the EQ-5D (*n* = 1,623). Similarly, Xie et al. [12] reported a difference of 0.04 in EQ-5D utilities between those not smoking 0.88 and those smoking 0.84 in the Medical Expenditure Panel Survey data (*n* = 39,680).

### Cost data

Costs gathered for the trial consisted of those from the GRx-related intervention and the programme set-up. The latter comprised the training costs for the intervention PSP, and PSP staff salary costs. The total training costs were amortised over all the participants in the intervention arm. Although referral to the GRx service was by researcher in the trial, the cost of GP referral is included. The cost per participant for Quitline was taken from their 2013 annual report.

Out-of-trial cost data by age, gender, and disease were estimated from the NZ HealthTracker database (Table 2). This database is a linkage system for all nationally collected health events, linked together by a personal unique identifier (National Health Index number). Each health event is linked to a unit resource cost, these include inpatient hospitalisations, outpatient attendance, laboratories, pharmaceuticals and (average capitation only from) general practice. Excess health system costs (healthcare costs above that for the healthy population) by age and gender, were calculated for the first year of diagnosis, subsequent annual costs (lung cancer only), and in the final six months of life for the disease states modelled [13].

Costs were reported in NZ$, net of Goods and Sales Tax, at 2012 base prices; they were inflated to 2012 price levels, where necessary, using the Consumer Price Index. Purchasing power parity exchange rates were used to convert currencies. The economic analysis took a NZ health system perspective.

### Markov model

The Markov model aimed to estimate the lifetime incremental costs and QALYs of adding the intervention to usual care. Trial costs and outcomes were used to parameterise this model.

In the base case, separate male and female cohorts were tracked from the age of 40 (the approximate average age of trial participants) until age 100. Two strategies were modeled; individuals either participated in usual care alone (Quitline) or the Fit2Quit intervention in addition to usual care.

**Fig. 1:**
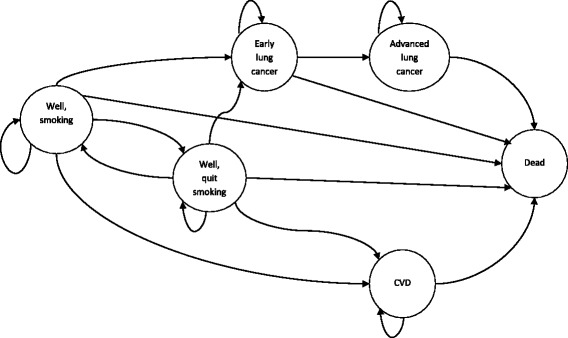
State transition diagram

Cohorts, starting in the ‘well’ state (Fig. 1), faced different probabilities of quitting smoking taken from the trial; relapse rates were included after continued abstinence at 24-week follow-up to 12 months, and in the three years beyond a successful quit attempt. The risk of developing lung cancer or CVD varied. Tunnel features have been built into the model for the lung cancer states to ensure that the risk of cancer progression or death is dependent upon the duration since diagnosis. For CVD, an assumption was made that an individual can have increased excess health system costs only in the year of diagnosis and death but they will have a permanently reduced HRQoL once they enter the CVD health state.

The Markov model is an extension of the smoking cessation model presented in Gordon et al. [14]. A CVD health state, NZ-specific epidemiological and cost data, different EQ-5D utility values and relapse pattern have been added. Readers are directed there for a more detailed explanation of the lung cancer component of the model, the structure of which remains unchanged.

### Analysis

The within-trial analysis was on an intention-to-treat basis. In the Markov model, only analyses comparing the adherent intervention group (defined as having 70% of calls delivered [7]) vs. the usual care group were performed. All participants with missing smoking status were considered to be smokers.

All future costs and health outcomes have been discounted at 3.5% per annum. In scenario analyses, the following were tested: a 0 and 5% discount rate for both future costs and QALYs; and changing the initial age of the cohort to 30, 50 and 60-years old.

The effects of variations to the parameters with plausible uncertainty in the base case were assessed using one-way sensitivity analyses (±2 standard deviations or 20% either way of the mean estimate), and simultaneously using probabilistic sensitivity analyses with Monte Carlo simulation and 1000 repetitions. Beta, log-normal, and gamma distributions were used for probabilities, relative risks, and costs, respectively (Table 2).

Incremental cost-effectiveness ratios (ICERs) were calculated to summarise the additional cost per unit of health benefit gained by switching from usual care to the intervention. For the trial analysis, the ICERs only included the programme costs, as additional costs to the health sector over those 24 weeks were not collected. All analyses were conducted using TreeAge Pro 2014 and Stata SE v11. Statistical tests were two-tailed and a 5% significance level was used. The Consolidated Health Economic Evaluation Reporting Standards (CHEERS) was adhered to [15].

## Results

### Within-trial cost-effectiveness

Descriptive data of the quit rates, HRQoL, leisure-time physical activity outcome measures and trial costs used for the cost-effectiveness analysis are presented in Table 3. Leisure-time physical activity showed a significant between-group change from baseline of 219 MET mins per week (adjusted difference) in favour of the intervention (*p* = 0.01). The average total cost per participant for the intervention group was NZ$ 623 and in the control arm was NZ$ 195.

**Table 3:** Trial outcomes and costs

*Outcomes*	*Intervention* (*n* = 455)	*Usual care* (*n* = 451)	*Difference*	
	Mean (se)	Mean (se)	Mean (95% CI)	
Quit rates at 24-week follow-up				
Point prevalence	23.1% (2.0%)	21.7% (1.9%)	1.3% (−4.1 to 6.8%)	
EQ-5D utility scores				
Baseline	0.792 (0.01)	0.800 (0.01)	−0.008 (−0.035 to 0.019)	
24-week follow-up	0.800 (0.01)	0.803 (0.01)	−0.002 (−0.029 to 0.025)	
Leisure-time physical activity (MET mins/week)				
Change from baseline	526 (69)	307 (66)	219 (53 to 386)	
*Costs*	*Quantity*	*Unit cost*	*2012 NZ$*	*Source*
Intervention, programme costs				
a) training for PSPs provided by investigators (trainer hours)	9	125	1,125	Trial
b) PSP salary incl. 25% overhead	2	75,000	150,000	Trial
Intervention, cost per participant (*n* = 455)				
a) PSP training costs per Fit2Quit participant			2.47	Trial
b) PSP salary			329.67	Trial
c) GP visit for Green Prescription			65.00	Estimate
d) pedometer	1	30.44	30.44	Trial
e) Quitline	1	195.33	195.33	[32]
		Total	622.91	
Usual care, Quitline cost per participant (*n* = 451)		Total	195.33	[32]

NZ$1 = US$0.68 = €0.53

*MET* Metabolic Equivalent of Task, *PSP* participant-support person

Over the 24-week follow-up, after adjustment for baseline HRQoL, there was a non-significant gain of 0.001 QALYs (95% CI: −0.006 to 0.008) in favour of the intervention, giving an ICER of NZ$ 451,000 per QALY gained. The ICER for 7-day point prevalence measured at 24-week follow-up was NZ$ 31,733 per smoker abstaining. Both values are unlikely to be considered cost-effective.

For the 52% who adhered to the intervention, the ICER for 7-day point prevalence measured at 24-week follow-up was NZ$ 3,991 per smoker abstaining – with 32.2% (se = 3.0%) abstaining in the adherent group.

### Lifetime cost-effectiveness for the adherent intervention group

In the Monte Carlo simulations, for a cohort with a starting age of 40 years, the adherent intervention participants compared with the control group gained 0.057 (males) and 0.068 (females) discounted QALYs till age 100 (Table 4). The ICERs were NZ$ 4,431 and NZ$ 2,909 per QALY gained for males and females respectively. At a threshold of NZ$ 20,000 per QALY gained, there was an 86% (males) and 90% (females) probability that the Fit2Quit intervention is cost-effective. In one-way sensitivity analyses of the base case, the main driver of uncertainty was the 12-month quit rates with other variables only having a minor impact.

**Table 4:** Lifetime cost-effectiveness for the adherent group

Adherent group	40-year-old male				40-year-old female			
*Base case*	*Intervention*	*Usual care*	*Difference*		*Intervention*	*Usual care*	*Difference*	
Mean cost per person *(NZ$)*	9,952	9,700	253		11,032	10,833	199	
QALYs gained per person	16.680	16.623	0.057		17.398	17.330	0.068	
ICER per QALY *(NZ$)*			4,431				2,909	
Probability cost effective^a^			0.860				0.902	
*Scenarios*	*Incr. costs (NZ$)*	*Incr. QALYs*	*ICER (NZ$)*	*Probability cost effective* ^a^	*Incr. costs (NZ$)*	*Incr. QALYs*	*ICER (NZ$)*	*Probability cost effective* ^a^
0% discount rate for costs & QALYs	−68	0.158	Dominant	0.894	−220	0.186	Dominant	0.893
5% discount rate for costs & QALYs	310	0.042	7,404	0.831	272	0.051	5,371	0.886
30-year-old cohort	307	0.055	5,633	0.879	275	0.061	4,521	0.895
50-year-old cohort	189	0.064	2,942	0.858	238	0.070	3,419	0.847
60-year-old cohort	128	0.065	1,976	0.777	203	0.069	2,962	0.814

^a^at NZ$ 20 k per QALY. NZ$1 = US$0.68 = €0.53

*ICER* incremental cost-effectiveness ratio, *QALY* quality-adjusted life-year

In scenario analyses, when varying the initial age of the cohort, discounted incremental QALYs were marginally higher, discounted incremental costs were lower and thus ICERs were more favourable for older cohorts. The probability that the intervention was cost-effective at NZ$ 20,000 per QALY gained ranged from 78 to 90% in these analyses.

## Discussion

To our knowledge, this is the first published cost-effectiveness analysis of an exercise-counselling intervention for smoking cessation. Positive effects were observed for those who adhered to the intervention, and for this subgroup it is likely to be cost-effective compared to usual care for increasing quit rates.

This study has several strengths; baseline prognostic factors were well-balanced, it is the largest study of its kind and the intervention was conservatively costed. For example, the intervention included an expensive NZ$ 30 Yamax SW-700 pedometer, which may not be necessary in practice as pedometer apps are now widely available for smartphones. The cost of GP referral was also included, although this was not necessary in the trial.

The study aimed to leverage existing national delivery services for both smoking cessation and physical activity promotion. The intervention was effective for increasing leisure-time physical activity. Physical activity is beneficial for a wide range of other health risks and outcomes such as depression, Type 2 diabetes, CVD and various cancers [16]. Therefore it is important that physically inactive or insufficiently active people who smoke are referred to programs to increase activity levels.

If the intervention could be targeted at those who would be willing to commit to seven or more intervention calls (the adherent group), the intervention may be more effective and probably cost-effective over 24 weeks compared to Quitline alone. Further, over the lifetime of the 40-year-old cohort (who were adherent to the intervention), Markov modelling suggests that the intervention may be cost-effective in improving HRQoL. These findings highlight the need to identify smokers who want to quit and *screen for readiness to exercise* to truly realise the benefits of such an approach and maximize smoking cessation outcomes: issues identified earlier [17].

There are limitations to this economic evaluation. First, verification of quit status was not undertaken due to the use of telephone-based assessments. Previous reviews of smoking cessation studies have shown that rates of misreporting of smoking abstinence are generally less than 5% [18]. Second, using the Markov model, the CVD and lung cancer states were mutually exclusive, and other (non-lung) cancers and respiratory diseases were not modeled. This may underestimate the impact of the intervention on future health outcomes and healthcare costs averted. Our reported QALY gains are below the benchmarks published by Stapleton and West [19]. While the costs of those other co-morbidities have not been modeled, their disutility may have been captured by the use of unadjusted population-based CVD and lung cancer utility scores. Third, the Markov model risk parameters for lung cancer were originally specified for heavy smokers (≥20 cigarettes/day) and may not apply to light smokers or those without the requisite pack-years. Fourth, and finally this economic evaluation using a lifetime horizon is limited to the adherent intervention subgroup: blanket provision for all smokers contacting Quitline is unlikely to be cost-effective. As this adherent subgroup was not prospectively defined, these results should be interpreted with caution and viewed as hypothesis generating. However, we wanted to explore what would make the intervention more cost-effective – higher quitting through greater adherence was a possibility.

Further research should be focused on identifying those who might be willing to commit to seven or more intervention calls. Improvements to the Fit2Quit intervention could include greater tailoring of the call schedule, increased face-to-face contact, and the provision of a support group. In addition, supplementing with electronic support, eg text messaging [20] or a smoking cessation smartphone app [21], is highly likely to improve cost-effectiveness [22]. Other options could include a commitment contract [23], or funding/provision through the workplace [24] and/or health insurance, where the productivity gains (not estimated here) and reduced claims may provide an incentive to intervene.

## Conclusion

If the exercise-counselling intervention could be targeted at those who would be willing to commit to seven or more intervention calls, it may be cost-effective compared to Quitline alone – improving both abstinence and leisure-time physical activity. Screening for readiness to exercise, an omission in the trial, would likely have helped to identify those potentially adherent.

## Abbreviations

CVD: Cardiovascular disease
EQ-5D: EuroQol five dimensions questionnaire
GRx: Green prescription
HRQoL: Health-related quality of life
ICER: Incremental cost-effectiveness ratio
MET: Metabolic equivalent of task
NZ: New Zealand
PPP: Purchasing power parity
PSP: Participant-support person
QALY: Quality-adjusted life-year
